# Qualitative European survey of patients with idiopathic pulmonary fibrosis: patients’ perspectives of the disease and treatment

**DOI:** 10.1186/s12890-016-0171-y

**Published:** 2016-01-14

**Authors:** Anne-Marie Russell, Elena Ripamonti, Carlo Vancheri

**Affiliations:** National Heart & Lung Institute, Imperial College & Royal Brompton Hospital, Respiratory Epidemiology, Occupational Medicine and Public Health, 1b Manresa Road, London, SW3 6LR UK; Elma Research S.R.L, Viale Tunisia 41, 20124 Milan, Italy; Regional Referral Centre for Rare Lung Diseases, Department of Clinical and Experimental Medicine, University of Catania, via S. Sofia 78, 95123 Catania, Italy

**Keywords:** Caregiver, Idiopathic pulmonary fibrosis, Impact, Information, Interview, Needs, Patients, Perspectives, Pirfenidone, Survey

## Abstract

**Background:**

‘Living with IPF and an exploration of Esbriet® – a new treatment’ was an exploratory, qualitative, real-world survey of European patients with idiopathic pulmonary fibrosis (IPF) who were receiving treatment with pirfenidone prior to its commercial availability. The aim of the survey was to probe the impact of IPF on patients’ quality of life; the role of healthcare professionals and caregivers; the information needs of both patients and their caregivers; and patients’ perceptions of pirfenidone as a new treatment option for IPF.

**Methods:**

Patients from the UK, Germany and Italy, with a diagnosis of IPF (duration >3 months), who were being treated with pirfenidone, were recruited from patient support groups, specialist centres and advocacy groups. Semi-structured, qualitative, in-depth patient interviews of 1-h duration were conducted by an independent researcher. Patients were initially asked about their experiences of living with IPF and then prompted to describe their experiences of taking pirfenidone. Techniques utilised included: the bubble-speech technique; the icon cards projective exercise; and the free association exercise. All interviews were transcribed and analysed by an independent researcher.

**Results:**

Forty-five patients (71 % male) were interviewed (mean age 68.5 years; mean time since diagnosis 3.5 years); 87 % of patients reported that diagnosis took >1 year. Patients reported that IPF had a significant physical and emotional impact on their quality of life. The beneficial role played by caregivers and interstitial lung disease specialist nurses (where available) was specifically highlighted. Although most patients were keen for information on IPF, this was often of poor quality, out of date, or in English only. Patients’ perceptions of pirfenidone were largely positive and associated with ‘hope’ but were also influenced by the level of side effects experienced.

**Conclusions:**

This survey highlights the impact of IPF on patients’ lives, and the need to adequately support both patients and their caregivers. These findings demonstrate the value of seeking patients’ perspectives of a chronic disease such as IPF and how this information can be used to guide improvements in care, to best support the needs of patients with this devastating condition.

**Electronic supplementary material:**

The online version of this article (doi:10.1186/s12890-016-0171-y) contains supplementary material, which is available to authorized users.

## Background

Idiopathic pulmonary fibrosis (IPF) is a chronic, progressive and irreversible interstitial lung disease (ILD) with a poor prognosis (2–5 years) [1–5]. IPF is predominant in men aged >50 years and, although idiopathic, risk factors may include gastroesophageal reflux, smoking and environmental or occupational exposures [1, 3].

IPF imposes limitations on many daily activities, necessitating a change in lifestyle. In addition to impacts on physical and social function, patients may also experience increased anxiety, depression and a reduced quality of life [6–8]. Increased sleep disruption and a poorer quality of sleep contributes to fatigue, further impacting the emotional and physical well-being of patients [9–11]. The impact of IPF affects not only patients but also caregivers, family and other members of patients’ support networks, all of whom have a role to play in helping to manage the burden of the disease [12, 13].

Until recently, the standard of care for patients with IPF was limited to oxygen therapy, pulmonary rehabilitation, palliative care and, in a small minority of patients, lung transplantation [1, 14]. Pirfenidone (Esbriet®), an antifibrotic drug with anti-inflammatory properties, was approved by the European Medicines Agency (EMA) in 2011 for the treatment of adult patients with IPF based on the favourable benefit-risk profile observed in Phase III clinical trials [15, 16]. The US Food and Drug Administration simultaneously approved pirfenidone and a tyrosine kinase inhibitor, nintedanib, for the treatment of patients with IPF in the USA, in October 2014 [17–20]; the EMA subsequently approved nintedanib in January 2015 [21].

Here, we report findings from ‘Living with IPF and an exploration of Esbriet® – a new treatment’, an exploratory, qualitative, real-world survey of European patients with IPF who were receiving treatment with pirfenidone prior to its commercial availability. The aims of this survey were to: explore patients’ experiences of living with IPF and how this impacted on their quality of life; determine the availability and impact of support systems, specifically that of healthcare professionals (HCPs) and caregivers; establish the information needs of both patients with IPF and their caregivers; and explore patients’ perceptions of pirfenidone as a new treatment option.

## Methods

### Design and patients

Patients were invited to enrol through patient support groups (UK), specialist centres (Italy) or an advocacy group (Germany). Eligible patients had a multidisciplinary team-confirmed diagnosis of IPF with disease duration >3 months. All patients were being treated with pirfenidone as part of a Named Patient Program (NPP) under the supervision of an ILD physician. Patients completed a screening questionnaire to verify their IPF diagnosis and that they were receiving treatment with pirfenidone. Forced vital capacity was not recorded as part of the questionnaire and, while the NPP was in principle limited to patients with mild-to-moderate disease, the decision to ultimately treat each patient was the responsibility of each participating physician, and, therefore, the inclusion of patients with more advanced disease cannot be excluded. All patients provided consent for their participation in the survey and for the future publication of anonymised findings from the survey.

### Interviews

Semi-structured, qualitative, in-depth patient interviews of 1-h duration were conducted by an independent researcher at a neutral venue or in the patient’s own home, according to preference. Interviews were conducted 1:1, although caregivers could be present at the patient’s request. Interviews were audio- and video-recorded (patients were asked not to identify themselves on tape). Patients’ experiences of living with IPF, as reported in this manuscript, were discussed initially; patients were then asked about their experiences of taking pirfenidone. The full interview discussion guide is provided (see Additional file 1). The interviews were designed to encourage frank, open discussion and to probe patients’ unmet needs and the emotional and rational elements driving their choices and behaviours. Techniques utilised in the interviews included: the bubble-speech technique, to probe patients’ expectations of their HCP and their level of satisfaction with the relationship; and the icon cards projective exercise and free association exercise to assess possible new formulations of pirfenidone.

### Analysis

All interviews were transcribed verbatim and, where necessary, translated into Italian; transcriptions were anonymised in accordance with local data protection laws and European Pharmaceutical Market Research Association Guidelines. Interview transcriptions were analysed by an independent researcher, and common themes and challenges were extracted and summarised. Disease awareness was determined by motivational and qualitative factors concerning the disease and knowledge of specific terminology. Side effects were reported as per the respondent’s spontaneous answers and were not analysed by a physician.

## Results

### Patients

Forty-five patients (71 % male) from three European countries (UK, Germany and Italy) were interviewed between 24 September and 19 October, 2012. All patients were invited to participate and approximately 80 % of the interviews were conducted with the caregiver present. Mean age was 68.5 years and mean time since diagnosis was 3.5 years at the time of interview. Patients reported that diagnosis frequently involved visits to several different HCPs, and 87 % of patients reported that diagnosis took more than 1 year. Approximately 90 % of patients were receiving oxygen therapy: 55 % used oxygen as needed, for a few hours of the day, while 35 % used continual oxygen therapy.

### Impact of IPF on quality of life

Approximately half of the patients reported that IPF had a significant negative impact on their quality of life. The most frequently reported physical symptom of IPF was fatigue (82 % of patients). Other physical symptoms reported by >50 % of patients included loss of appetite (typically in response to feelings of nausea), difficulty in lifting objects (eg, grocery bags), and continuous phlegm and coughing throughout the day.

*“Even the simplest things are difficult for me” (Patient – Italy)*

Patients reported that the physical impact of IPF often took an emotional toll, leaving them feeling depressed and often without the emotional strength to fight disease progression. The primary emotional concern reported by patients was fear of the disease progression and its impact on their future (72 % of patients). In addition, 36 % of patients reported feeling frustrated and angry, due to a lack of self-acceptance of their disease and other people’s poor knowledge of IPF. Patients also reported feeling isolated (18 %), causing them to withdraw from social relationships.

*“I feel really sad; before I was a very cheerful person” (Patient – Germany)*

Patients reported that IPF also impacted on family life. Some patients perceived themselves to be a burden and felt reliant on their family, leading to additional frustration for the patient. Most female patients (90 %) reported a loss of identity as the main family support figure, and some of the male patients (15 %) reported a detrimental impact of IPF on their sex lives, both physically and emotionally. No other gender differences were identified.

*“I don’t know what type of life I can offer my wife, I don’t feel like a man anymore, because of me I am forcing restrictions on myself and other people” (Patient – Italy)*

*“My family checks everything I do, I don’t feel free” (Patient - UK)*

The initiation of oxygen treatment marked an important stage in disease progression for all patients, due to the perceived limitations it placed on their freedom. Oxygen therapy was associated with less hope for the future and more feelings of shame as their condition became externally visible to others.

*“I’m always hooked up to the oxygen, I have a range of movement of 10 m” (Patient – Italy)*

### The role of HCPs and caregivers

In the UK, 90 % of patients reported that an ILD specialist nurse was their main medical contact for IPF healthcare. Nurses provided information on IPF, patient support organisations and lifestyle advice, as well as helping patients to access medication and manage side effects.

*“She [the nurse] is everything for me, she is a shoulder to cry on, she answers any question I ask her, I hope she never leaves!” (Patient – UK)*

In Germany, patients reported that physicians were their main contact, particularly concerning treatment efficacy and side effects, whereas nurses played a more marginal role in their care. In Italy, patients perceived IPF as a rare condition requiring specialist knowledge; they reported that nurses were not involved in their care and that they generally interacted exclusively with their physician.

*“I am one of many patients; it is not easy to build a relationship” (Patient –Germany)*

*“The doctor is my reference; he is the person I call, definitely not the nurse who does not know much” (Patient – Italy)*

Most patients reported feeling that they lacked the psychological support necessary to face their IPF-related difficulties, with only one-fifth of patients having received professional psychological support.

*“The doctors helped me, but I have never received any psychological support, I really need it” (Patient – Germany)*

More than half of the patients felt they needed a caregiver in their household. Patients reported several important roles that were fulfilled by caregivers, including reminding patients to take medications, accompanying patients to medical appointments and researching information on IPF. Many caregivers present during the interviews demonstrated a greater knowledge of IPF than the patient.

### Information needs of patients and their caregivers

The level of disease awareness varied widely amongst patients, but approximately one-third of patients felt inadequately aware of or informed about IPF. Patients in the UK and Germany largely understood the severity of their IPF and their prognosis, as well as the available treatments, whereas patients in Italy generally reported being less well-informed about IPF and mostly relied only on information provided by their physician.

*“No idea about the name of this disease, I call it lung infection” (Patient –Italy)*

Most patients (74 %) reported searching for information about IPF. However, many patients described issues with what they found, including poor-quality and out-of-date information, which was only available in English.

*“It’s imperative to have information about this disease” (Patient – Germany)*

Patients in the UK and Germany preferred to receive information about IPF online – most frequently via the Pulmonary Fibrosis Foundation, British Lung Foundation (BLF) and LungenFibrose websites – whereas Italian patients preferred printed materials. The main areas of patient interest included information on IPF, the use of pirfenidone, and updates on new research and therapy (Table 1). Emerging needs spontaneously reported by patients concerned a lack of expert advice relating to lifestyle (51 %), diet (38 %), oxygen treatment (38 %) and physical exercise (18 %). Many patients reported that they would like to receive practical information about lifestyle changes that would help them manage their disease. Patients also expressed an interest in knowing how many other people in their area were affected by IPF and wanted the general public to be better informed about the disease.

**Table 1:** Patients with IPF indicated clear needs for information in three areas

IPF	Pirfenidone	Updates on new research and therapies
Greater knowledge about IPF, the causes of the disease and the number of people affected	More data regarding the efficacy and tolerability of pirfenidone, including how to practically manage tolerability issues	Information on the new drugs that are being studied for IPF, including results from clinical trials

*IPF* idiopathic pulmonary fibrosis

Caregivers reported feeling inadequately prepared for the caregiving role. Caregivers stressed a need to receive adequate psychological support to accept the disease and better relate to the patient; a need for strategies to manage the everyday life of a patient with IPF; and a need for more background information on IPF.

*“It is difficult for us to help them, we would like to have more information about what we should do at home” (Caregiver – UK)*

### Patients’ perceptions of pirfenidone

Patients’ perceptions of pirfenidone were largely positive, scoring 7.4 on a scale of 0 (not satisfied) to 10 (very satisfied). Satisfaction was influenced by the level of side effects experienced by patients, with an average satisfaction score of 6.1 (*n* = 25) for patients with side effects and 8.3 (*n* = 20) for patients with no side effects. Side effects, including nausea and photosensitivity, were reported to have a negative psychological impact on patients who perceived little awareness of their treatment working. Patients with side effects also reported a general deterioration in their quality of life, which they perceived to be related to pirfenidone. Furthermore, if a patient was experiencing side effects, they often attributed all of these effects to pirfenidone, even if any correlation between the two was unconfirmed.

*‘I have so many problems, such as nausea, itchy legs and backache’ (Patient – Germany)*

Patients without side effects reported feeling that their condition had stabilised with the use of pirfenidone, which in turn gave them a sense of hope and the feeling they were being taken care of by HCPs. Patients reported feeling reassured when HCPs confirmed their disease had stabilised (according to clinical parameters), which gave them greater confidence for the future.

*“I feel I’m taking something that can really halt this disease and make me live longer” (Patient – Germany)*

*“Ever since I started taking pirfenidone, I feel more protected” (Patient – Italy)*

## Discussion and conclusions

IPF is a devastating disorder and the findings of this qualitative survey of UK, German and Italian patients with IPF add to the existing body of knowledge regarding the substantial impact IPF has on the lives of patients and their caregivers [6–11]. In agreement with recent literature [12, 22], these findings also identify the need to more adequately support patients with IPF in terms of disease awareness, lifestyle management, psychological support and management of medication side effects, as well as emphasising the important role played by the HCP in having primary contact with the patient and their family.

Approximately one-third of patients in the survey were identified as being unaware of and/or uninformed about IPF, and most patients wanted a better understanding of the disease. However, sources of information on IPF were frequently of poor quality, out of date or not available in the patients’ native language. In the UK and Germany, patients predominantly used online sources when looking for further information about their condition, while Italian patients reported a preference for printed materials; however, it was unclear whether this was actually due to preference or to a lack of web-based IPF information in Italian. In general, however, when diagnosed with a chronic disease such as IPF, many patients now turn to online sources of health information as this allows them a degree of autonomy, and the ability to examine and digest information at their leisure [23]. The findings of this study support the need for good-quality, freely available, online information on IPF in multiple languages, to support patients in learning more about the practical management of their disease. It is also interesting to note that many patients expressed a desire for better understanding of IPF among the general public. The recent US EXPLORE survey found that patients with IPF often felt isolated, embarrassed and stigmatised [24], and patients with other chronic respiratory diseases, such as chronic obstructive pulmonary disease, often also report feeling stigmatised [25].

The findings of this survey also emphasise the important role played by the HCP who is the primary contact for a patient, whether they are a physician or a specialist nurse. In the UK, specialist nurses were reported to play an important role in supporting patients with many aspects of disease management and patients clearly felt this was beneficial. However, the UK is quite unique in having advanced ILD specialist nurses who are able to play a significant role in helping patients understand the disease and coordinate their care [22]. In a recent report by the BLF, 50 % of English healthcare trusts reported allocating an ILD specialist nurse within 6 months of a patient’s diagnosis [22]. Furthermore, UK clinical guidelines state that all patients with IPF should have an ILD specialist nurse allocated to them and that any centre that wants specialist status must employ a specialist ILD nurse [26]. UK clinical guidelines also recommend that the ILD specialist nurse should provide accurate and clear information to patients and their families, regarding diagnosis and management of IPF, and support the patient at all stages of the care pathway to facilitate transitions in care and advise on symptom management [26, 27]. In Germany and Italy, nurses were less involved in patient care. Specialist nurses for IPF care may warrant consideration by German and Italian healthcare authorities due to their ability to provide a holistic programme of care.

Caregivers were present in 80 % of the interviews conducted for this survey and provided a useful source of information. However, it should be acknowledged that the presence of caregivers during the interview may have inhibited responses from some patients. Nevertheless, the findings of this survey support the difficulties faced by caregivers in looking after patients with IPF and are in agreement with the literature. The caregivers of patients with IPF have reported hardships throughout the course of a loved one’s disease, including emotional devastation at the initial diagnosis and difficulties living with a loved one because of their limitations [28, 29]. Furthermore, providing additional support to caregivers of patients with IPF has been demonstrated to provide some positive benefits. For example, caregivers who attended a 6-week nurse-led group intervention programme on IPF management, alongside their loved ones with IPF, reported significantly lower stress levels post-intervention compared with those who did not attend the intervention programme [29].

Pirfenidone was the first medication approved for the treatment of European patients with IPF, and the positive feedback reported in this survey suggests it provides hope and reassurance to patients. However, the findings of this survey also highlight the need for guidance on how to identify and mitigate the side effects experienced by some patients during treatment with pirfenidone, such as the nausea and loss of appetite reported as symptoms of IPF in this study, which may have been related to pirfenidone therapy [16, 17] rather than to IPF itself. It should be noted that this survey was conducted in 2012, prior to the launch of pirfenidone in some European countries (including the UK). At this time, HCPs would have been less familiar with pirfenidone and its side-effect profile than they are at present. Recent reports from real-world studies suggest that the tolerability issues with pirfenidone can be well managed, with no new safety signals identified versus the clinical trial programme [16, 17, 30, 31]. However, there remains a need to provide patients with lifestyle guidance when they are taking pirfenidone to minimise the risk of treatment side effects. Some strategies that have proved successful include using sun protection to avoid photosensitive skin reactions and taking pirfenidone during or after a meal to alleviate effects on gastric motility [32].

While this survey reports the impact IPF has on patients’ lives and supports the need to address the unmet needs of patients’ and their caregivers, several limitations must be addressed. The survey was not designed to exclusively evaluate patients’ experiences of IPF. No formal measure of quality of life was implemented and patients were not randomly selected for participation in the survey. Furthermore, the sample size was small, although this is typical of other surveys that have been conducted in patients with IPF [2, 6, 11].

### Developments in IPF management and disease awareness

As a result of surveys such as this one, many aspects of care for patients with IPF in Europe have changed since 2012. In 2013, the patient support initiative ‘IPF Care’ was established in eight European countries, to provide help and education for patients treated with pirfenidone through frequent discussions with ILD specialist nurses. The benefits of the programme are already receiving recognition, such as in the UK where 66 % of patients who reported an adverse event during the ‘IPF Care’ programme remained on maintenance therapy [13], and in Austria, where 96 % of patients who started pirfenidone with the support of ‘IPF Care’ remained on treatment for ≥3 months compared with only 64 % of patients who were not enrolled in the programme [13]. The success of ‘IPF Care’ demonstrates the value that patient support programmes can offer as a tool for complementing and enhancing the support provided by healthcare systems, and as a forum within which to discuss patients’ worries. Other initiatives include IPF World Week, established in 2012, to raise awareness of the disease through its ‘Blowing Bubbles’ campaign in several European countries [33], and the European IPF Patient Charter, established to support equal access to IPF treatment and care standards in Europe [34].

In the UK specifically, there have been many advances in IPF, including the launch of two patient advocacy groups (Pulmonary Fibrosis Trust in 2011 [35] and Action for Pulmonary Fibrosis in 2013 [36]), which provide information and support to patients, and the launch of a clinical pathway for IPF in early 2015 [37]. Furthermore, the BLF has supported the development of many projects, including an IPF Patient Charter (supported by multidisciplinary IPF experts, patients and caregivers) [38], a survey evaluating patients' experiences in England [22] and numerous sources of patient information. The BLF is also contemplating the development testing the feasability of a personal organiser for patients with IPF in the UK.

In Italy, general awareness of IPF is slowly improving; for example, there are several patient associations, including RespiRARE in Sicily, dedicated to rare lung diseases [39] and Ama Fuori dal Buio, which is very active in supporting patients and providing them with disease information [40]. The Observatory for Rare Disease conducted a successful social-media campaign inviting people to post pictures or videos of them trying not to breathe, to raise awareness of IPF [41], and in 2013, a patient- and caregiver-specific website was launched with disease information in Italian [42]. Italian clinical guidelines for IPF were also recently published [43]. In Germany, the patient association Lungenfibrose e.V is also very active in supporting patients and providing disease information [44], and clinical guidelines for IPF were launched in 2013 [45].

### Future directions in IPF management and disease awareness

Nevertheless, more work is still needed to improve the diagnosis and management of IPF. In this study, most patients reported a delayed diagnosis and this is not uncommon for IPF and other rare diseases [46], as demonstrated by the results of the BLF’s recent survey where many patients with IPF in England reported struggling to obtain a diagnosis [22]. Patients in the BLF survey also reported not having access to information about IPF that they could understand, having to navigate their own care pathway, and a lack of access to an ILD specialist nurse. We should, therefore, continue to seek each patient’s perspective on their disease and treatment, so that the information and care they receive can be tailored to their specific needs, thereby providing the support they and their families require to live with IPF.

## Additional file

Additional file 1:
**Discussion guide: Living with IPF and an exploration of Esbriet® – a new treatment (1 h).** (DOCX 21 kb)

## Abbreviations

BLF: British Lung Foundation
EMA: European Medicines Agency
HCP: healthcare professional
ILD: interstitial lung disease
IPF: idiopathic pulmonary fibrosis
NPP: Named Patient Program
