# Supplementary material for: Qualitative European survey of patients with idiopathic pulmonary fibrosis: patients’ perspectives of the disease and treatment
Source: BMC Pulm Med. 2016 Jan 14;16:10. doi: 10.1186/s12890-016-0171-y (PMC4712607; doi:10.1186/s12890-016-0171-y)
Supplement: Additional file 1: — Discussion guide: Living with IPF and an exploration of Esbriet® – a new treatment (1 h). (DOCX 21 kb) [file 12890_2016_171_MOESM1_ESM.docx]

## Additional file 1

### Discussion guide: Living with IPF and an exploration of Esbriet^®^ – a new treatment (1 hour)

1. **Introduction of the topic**
   - Introduce yourself/present Elma Research
   - Explain the aims of the research
     - “*Today we are going to deal with idiopathic pulmonary fibrosis (IPF), in particular I will ask you about your experience and opinion of some specific drugs…”*
   - Our discussion will last approximately 1 hour
   - We comply with all relevant codes of conduct and legislation for confidentiality in market research, including EphMRA and Esomar
   - Explain the use of audio and video recordings – reassure the patient about the guarantee of anonymity
   - Confidentiality/no promotional aims/there are no right or wrong answers
   - Elma Research adheres to the normative on adverse events
     - *“We are asked to pass on to our client details of adverse events that are mentioned during the course of the interviews for this market research. The answers you give during this interview will obviously be protected and treated in confidence. However, should you make a reference to any adverse event, we will need to report this to the company’s pharmacovigilance department even if it has already been directly reported by your doctor. In such a situation, we will read you the informative, either during or at the end of the interview, to obtain more information about the adverse event.”*
   - Make sure the patient has signed the Privacy Form before proceeding
2. **Presentation of the interviewee: the patient and IPF (aim: make the interviewee feel at home)**
   - First of all could you tell me your first name and a little about yourself
     - Your family, your age/your children’s ages, your lifestyle
     - Do you work yes/no, type of work
     - Three words that describe you…
   - Talking about IPF*,* when did the first signs of the disease start?
   - When did you find out that you had the disease? Who made the diagnosis? Whom did you go to when you had the first symptoms? Who started you on therapy?
   - What did you think when you found out you were suffering from IPF? How did you feel (could you describe your feelings)? What were you most worried about? Did you have any doubts?
   - What were you afraid of at that moment?
   - What happened after that? Could you tell me what happened?
   - How would you describe your health today? What are the main problems? What other disease do you suffer from if any (probe at a high level, don’t go into detail)? What are your main worries?
   - What is the impact of IPF on your daily life?
   - What are the greatest limitations you have in your everyday life due to IPF?
     - Physically? (Probe on concrete examples)
     - Emotionally?
   - Is there anything that you had to give up because of IPF? ie physical activities, what, when, why..?
   - What are the concerns you have about the future, if any? What is the major concern for you?
   - Do you have any further issues?
   - Where do you get information about IPF (internet, doctor, other patients)?
   - Are you in touch with any other patients with IPF? Why?
   - If not, would you like to be in touch with other patients with IPF?
   - Do you belong to any IPF patient organisation?
   - Do you have caregiver support?
   - If yes, what role do they play? Probe (information about IPF, therapies, getting the drug from pharmacy/hospital, remind to take the medication)?
   - What role do nurses play? Explain? (Provide info on treatments, disease, etc?)
3. **Current Esbriet**^®^ **formulation**
   - What medications have you ever taken for IPF?
   - What drug therapy are you taking for IPF?
   - When did you start taking Esbriet^®^? How long have you been taking it?

**The start of Esbriet**^®^ **therapy and the degree of satisfaction**

**Bubble-speech technique**: hand the interviewee the cartoon with the speech bubbles

- In the sheet I am going to give you, you will find a cartoon of you with your doctor. Could you fill in the ‘bubbles’ describing what your doctor said to you when he/she prescribed Esbriet^®^ for the first time. Then also write what you asked your doctor and, lastly, what you were thinking but did not say to your doctor.

Collect the comments about what has been written.

- - When your doctor first told you about Esbriet^®^, how did they explain it to you (if at all)?

MODERATOR:

- Probe the exact language the HCPs used to describe IPF
- Also probe what language patients say their HCP used to explain what Esbriet^®^ can do
- Probe on safety, efficacy
- Listen for price but do not probe
  - Do you feel your doctor’s description was good?
  - Would you like to have more or less information about Esbriet^®^?
  - What has changed for you since you started taking Esbriet^®^?
    - Which feeling? What you want to say…
    - And from a physical point of view? What has it changed?
  - Were you uncertain about anything when you started the treatment with Esbriet^®^? What? About what exactly?

MODERATOR: Try to understand (without asking directly) if it was strange that the drug is a pill versus an inhaled treatment

- - Did Esbriet^®^ change your daily life in anyway? In what way?
  - What if anything has improved? What if anything has become worse?
  - What is your degree of satisfaction with Esbriet^®^?
    - Are there any bothersome/problematic aspects of taking Esbriet^®^? Why is that?
    - Have you been told about side effects/safety? What has your physician told you about that/what to expect?
    - Have you ever had any side effects with Esbriet^®^? Probe on type? And how they have been managed?
    - What would you like to change? Why is that?
    - If you could improve anything about this drug what would you improve?

**Current Esbriet**^®^ **formulation**

- - (For Germany only) How do you find the packaging of Esbriet^®^ pill to be? What, if any, problems do you have with the packaging?
  - How many Esbriet^®^ tablets do you take a day?
  - When you first started taking Esbriet^®^, how many pills did you take? First week? Second week? Now?

MODERATOR: Probe if the patient is not currently taking 9 pills a day and ask why

- - How do you find taking 9 pills per day? If difficulties are mentioned probe for details
  - What impact, if any, does taking 9 pills per day have on your daily life? When the doctor said you should take Esbriet^®^, what did he/she say about taking the product?
    - What instructions did he/she give you?
  - Did you have any questions?
  - Has your doctor every told you to ‘step down’ the dose of Esbriet^®^ 9 pills a day? Why? Did it work to avoid side effects?

*(For Germany only)* MODERATOR: If a patient has reduced their dose ask:

- - - What package do you use? (Note that there are 3 packages: 1 week [3 pills a day], 2 week [6 pills a day] and 4 week [9 pills a day] packages)
  - Have you ever forgotten to take the tablets? How often has this happened?
  - Did you tell your doctor?
    - *(For Germany only)* Does the Esbriet^®^ packaging help you remember to take your Esbriet^®^ pills on time? If no, what would help you remember? When do you go back to your doctor for refills of Esbriet^®^ (monthly, every 3 months?)
- Do you ever interact with a nurse with regards to Esbriet^®^?

**4.** **The new formulations**

MODERATOR SHOW FORMULATION 1: *“Now I will show and explain to you a new possible Esbriet^®^ formulation”* (rotate presentation of formulations)

- - Record the spontaneous reactions
  - The first adjective that comes to mind to describe it

**Free association exercise**: new Esbriet^®^ formulation

MODERATOR: *“Now I will show you a list of* ***adjectives and words****, please choose the ones that represent the way you feel when thinking about this new Esbriet*^®^ *formulation”*

- - Why did you choose these words? What do you mean?
  - What do you think about this new formulation?
  - What are the advantages compared to the current formulation?
  - What problems would the new formulation be able to solve?
  - If your doctor proposed this new formulation to you what questions would you ask him/her? What would you like to know?

NOTE FOR THE MODERATOR: repeat the same questions for formulations 2 and 3

5. **The favourite formulation**

MODERATOR: *“Now that you have seen the three new possible Esbriet*^®^ *formulations, which one would you choose?”*

- - Why this formulation?
  - What are the advantages compared to the other formulations?
  - Would you prefer this formulation to your current formulation?
  - Probe on why?

**Icon cards projective exercise**: new favourite Esbriet^®^ formulation

MODERATOR: *“Now I will show you the blob tree, could you tell me which picture best represents the new Esbriet*^®^ *formulation you prefer?”*

- - Could you help me to understand what this picture represents exactly, what is it trying to communicate?

**6. Adherence programmes**

MODERATOR:

How do you remember all your different medicines?

Do you keep a record of the medicines you take, your clinic appointments etc?

Who helps you?

How often do you speak to your nurse/clinic? Do they have time to answer all your questions?

Do your partner/family members/caregivers speak to the clinic team?

What questions/information are they good with, what questions are they not so good at helping with?

What printed or online information did they give you? Can you remember any particular booklets or websites that you found extremely helpful?

Do they help you find other support outside the clinic, for example, system benefits, local support groups?

Does your clinic team ask you to track your progress?

When you are breathless? And how you manage it?

Your saliva and how it changes with how you are feeling

Have you received information about what food to eat, managing your weight etc?

Have you been told about staying active?

What about travelling? Do/can you still travel? How does your condition and treatment affect this? Would you like help with travel?

Is there any other support or information you can think of that would be helpful?
